# EM-Gaze: eye context correlation and metric learning for gaze estimation

**DOI:** 10.1186/s42492-023-00135-6

**Published:** 2023-05-05

**Authors:** Jinchao Zhou, Guoan Li, Feng Shi, Xiaoyan Guo, Pengfei Wan, Miao Wang

**Affiliations:** 1 State Key Laboratory of Virtual Reality Technology and Systems, Beihang University, Beijing, 100191 China; 2 Kuaishou Technology, Beijing, 100085 China

**Keywords:** Computer vision, Gaze estimation, Metric learning, Attention, Multi-task learning

## Abstract

In recent years, deep learning techniques have been used to estimate gaze—a significant task in computer vision and human-computer interaction. Previous studies have made significant achievements in predicting 2D or 3D gazes from monocular face images. This study presents a deep neural network for 2D gaze estimation on mobile devices. It achieves state-of-the-art 2D gaze point regression error, while significantly improving gaze classification error on quadrant divisions of the display. To this end, an efficient attention-based module that correlates and fuses the left and right eye contextual features is first proposed to improve gaze point regression performance. Subsequently, through a unified perspective for gaze estimation, metric learning for gaze classification on quadrant divisions is incorporated as additional supervision. Consequently, both gaze point regression and quadrant classification performances are improved. The experiments demonstrate that the proposed method outperforms existing gaze-estimation methods on the GazeCapture and MPIIFaceGaze datasets.

## Introduction

Human gaze contains the information of interest, intention, mental state, and concentration level of a person. It is critical to estimate gaze using computational models. Over the past decades, various gaze-estimation methods, which can be categorized into model- and appearance-based, have been proposed. Model-based methods typically require specific devices to build an eye model and track the gaze. Stable and accurate gaze can be tracked once person-specific calibration results are provided. However, owing to the requirement of specific devices such as infrared lights, their scalability on commodity mobile devices are limited. With the increasing use of mobile phones and tablets, gaze estimation from monocular face images have attracted more attention in the fields of computer vision and human–computer interaction. Appearance-based methods simply use monocular images as inputs, which facilitates the application of gaze estimation in daily life.

The introduction of deep neural networks into gaze estimation [1] has improved appearance-based methods. Krafka et al. [2] proposed taking the face image along with cropped eye images as network inputs and constructed a large dataset of face images and corresponding gazes, collected via daily mobile devices. Although recent studies [3] have significantly progressed in gaze estimation on mobile devices with multiple calibrations, Bao et al. [4] improved the gaze-estimation performance on the calibration-free setting, which is more suitable for devices with high real-time requirements. There remains a gap between estimation results and practical applications, which is primarily caused by the relatively large estimation error (approximately 1.6 cm) over the mobile device screen size (e.g., 7.57 cm of iPhone 11). For example, in determining whether a user is looking at a quadrant division of the mobile phone screen, gaze point regression errors can cause up to 43% wrong predictions.

**Fig. 1:**
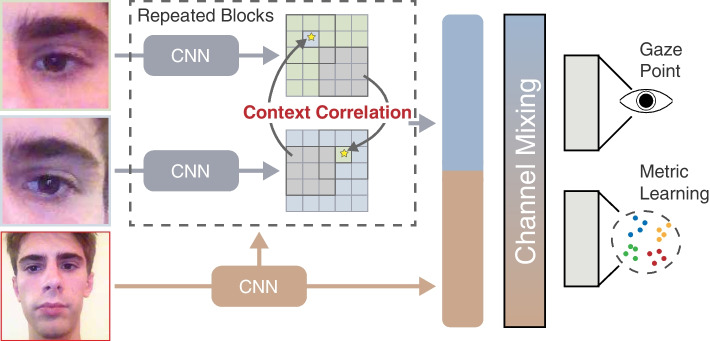
Overview of the proposed method. The goal is to estimate 2D gaze from an input face image. CCBs are proposed to efficiently correlate eye features, and employ metric learning to optimize gaze-feature embedding

In this study, a neural network that uses gaze classification on quadrant screen divisions as additional supervision for mobile gaze regression is proposed. This study first explores mutual connections between eye features using context correlation blocks (CCBs), and fuse eye and facial features using light-weight channel-mixing layers. Moreover, metric learning is incorporated into the regression task for classification and the effectiveness is demonstrated through gaze regression and classification results. Figure 1 shows a high-level overview of the proposed method. The main contributions of this study are as follows.

- A novel CCB that correlates contexts between eyes for deep gaze-feature extraction.
- A metric learning strategy based on gaze classification on quadrants for gaze feature-embedding optimization.
- A neural network EM-Gaze that achieves state-of-the-art performances on unconstrained gaze-estimation datasets.

### Gaze estimation

With the rapid development of mobile devices, reconstructing 3D human faces in a simple, accurate, and practical manner has become a critical task for computer vision and human–machine interaction. Gaze estimation is essentially related to facial expression [5] and face reconstruction [6], however, it could be more independent owing to the needs of specific data acquisition. A recent survey [7] has carefully discussed this field. Over the past decades, several gaze-estimation methods, which can be categorized into model- and appearance-based, have been proposed [8, 9]. Orthogonal to the model- and appearance-based methods from facial or eye images, gaze estimation from observed scenes [10, 11] is also a crucial research direction, especially in virtual reality scenarios. This study briefly reviews some representative model- and appearance-based methods.

Model-based methods rely on hand-crafted feature extraction to construct a geometric eye model and obtain robust gaze-estimation results [12]. Eye features are obtained by near-infrared corneal reflections [13], pupil center [14], and iris contours [15, 16]. Although most model-based methods have stable performances [17], they require additional apparatus, such as infrared lights or stereo cameras, in restricted environments.

Appearance-based methods formulate gaze estimation as a regression problem that takes eye or face images as inputs and predicts the 3D gaze direction or 2D gaze point from the images. These methods only require a monocular camera to capture the user’s facial images, thus it can be easily applied to mobile phones. To learn a general regression function for gaze estimation, methods such as adaptive linear regression [18], Gaussian process regression [19], and dimension reduction technique [20] have been proposed. However, such methods fail to fit high-dimensional non-linear mapping functions. Recently, the deep neural network has made significant achievements in various computer-vision tasks and has demonstrated its value in gaze estimation. Zhang et al. [1] proposed a convolutional neural network to estimate 3D gaze direction from eye images based on LeNet [21]. Yu et al. [22] proposed to estimate eye gaze and face landmarks simultaneously. Fischer et al. [23] used a VGG-16 network [24] to extract deep eye features. Cheng et al. [25] explored the asymmetry between left and right eyes for gaze estimation. Park et al. [26] proposed a novel encoder-decoder-based framework with meta-learning for the few-shot gaze-estimation task. Recent methods [27, 28] have achieved good performances on 3D gaze estimation. Krafka et al. [2] for the first time employed the convolutional neural network to estimate 2D gazes and proposed a large-scale 2D gaze dataset called GazeCapture. He et al. [3] used light-weight networks to achieve similar accuracy with higher efficiency on mobile phones. Lemley et al. [29] further improved the efficiency of gaze estimation with the simplified convolutional neural network on low-quality devices. EyeNeRF provides an efficient method for generating large eye datasets, which may benefit gaze estimation [30]. Recently, a novel adaptive feature fusion network AFF-Net was proposed [4]. It achieves state-of-the-art gaze point estimation results on the GazeCapture [2] and MPIIFaceGaze [31] datasets.

### Softmax-based metric learning

Metric learning has demonstrated its advantages in face recognition [32] and person re-identification [33] tasks. Deep metric learning can better discover the intrinsic relationships between features through feature-distance mapping, which can significantly improve classification performance in vision tasks. Softmax-based methods aim to apply different embedding distances on the calculation of logits for discriminating high variance samples in the face recognition task, which is similar to the gaze point classification task. For instance, Liu et al. [34] proposed a large margin softmax (L-Softmax) loss by adding angular constraints, which was later improved with a weight normalization scheme [35]. Wang et al. [36] defined the decision margin in the cosine space that achieved state-of-the-art performance based on a survey of metric learning [37]. Softmax-based metric learning for gaze point classification on quadrant regions can further optimize gaze-feature embedding and facilitate the discrimination of different gazes.

### Attention mechanism in vision tasks

Attention mechanisms have been widely used in natural language-processing tasks, and numerous works are also devoted to adapting the attention mechanism to computer-vision tasks. SENet [38], a representative attention-based architecture, explores the attention and gating mechanisms between deep local features. With the proposal of Transformer [39], attention has been proven to perform better than convolutional neural networks in certain vision tasks because Transformer has a better global perception of the entire image. ViT [40] completely adopts the Transformer structure into vision pipeline and achieves better performance. Li et al. [41] proposed a unified building block by introducing 3 × 3 convolution into attention to obtain fine-grained attention maps. Whereas the above methods primarily focus on self-attention of a single image, certain studies performed feature correlation between images. Recently, Chen et al. [42] combined cross-attention with Transformer to further improve the classification performance. Attention mechanism has also been widely used for several tasks, such as behavior recognition [43] and segmentation [44]. This study introduces contextual attention into the cross-attention paradigm and proposes CCBs in the EM-Gaze network.

## Methods

In this section, the technical details of the EM-Gaze network that explicitly considers the correlations between eye contextual features and leverages metric learning for quadrant division-aware supervision are elaborated.

### Overview

Given a face image x ∈ R^H×W ×3^ (H and W are the height and width of the image), the goal of 2D gaze estimation is to predict a 2D gaze vector y ∈ R^2^ that indicates the physical position on the screen, measured from the top-left corner in centimeters. In the proposed method, as a side-product, the corresponding quadrant division label q ∈ {1, 2, 3, 4} of the screen within which the gaze is located is predicted. A two-stream collaborative architecture for computing context correlated features for left and right eyes is proposed under the guidance of facial features. The network is supervised by normally used gaze-regression loss and the proposed gaze-classification loss on quadrant divisions.

The proposed method first extracts facial features from the input image to guide left and right eye feature extraction individually. Subsequently, contextual features are iteratively computed and correlated for the left and right eyes through CCBs, which adaptively assign shared attention weights to the eye features. Facial and correlated eye features are then concatenated and processed by channel-mixing layers for long-distance feature channel fusion. From the mixing layer, the 2D gaze point is predicted using a fully connected layer, supervised by a regression loss. Additionally, the network is supervised by incorporating metric learning for gaze classification on quadrant divisions.

### Two-stream collaborative architecture

In appearance-based approaches, eye features are computed from an input image to regress the gaze point. Krafka et al. [2] and Zhang et al. [31] observed that facial features, such as relative eye positions on the face and head pose, can provide additional cues for gaze estimation. Previous studies have used eye features in different ways. Cheng et al. [25] demonstrated that the two eyes have different confidence of accuracy and proposed the ARE-Net that adaptively adjusts the weights for eyes. CA-Net uses both eyes and the face to model multi-scale eye representation with a coarse-to-fine strategy for gaze estimation [45]. Bao et al. [4] introduced adaptive group normalization (AdaGN) to re-calibrate eye features based on facial features and used SELayers to adaptively fuse concatenated eye features and facial features. Mutual information between the left and right eyes’ connection is not fully exploited. Existing attention models weight eye features by either cooperating facial information or calculating self-attention using single eye image. However, this study enhances the mutual connection between eyes with iterative correlations between eye contexts using a two-stream collaborative architecture.

### The network

**Fig. 2:**
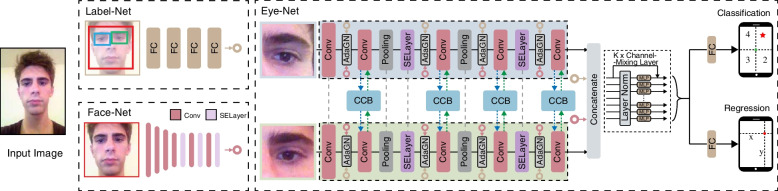
Structure of the proposed EM-Gaze network. Given an input face image, facial features are extracted by Label-Net and Face-Net. Eye-Net takes the left eye image and flips right eye image as inputs, extracts eye features under the guidance of facial features, and iteratively correlates two-eye features using the proposed CCBs. Concatenated eye and facial features are fed into channel-mixing layers to obtain the gaze feature. Finally, fully-connected layers are employed to estimate 2D gaze position and quadrant division-based classification

EM-Gaze contains three closely related sub-networks: Label-Net, Face-Net, and Eye-Net (Fig. 2). Label-Net takes detected face and eye bounding box labels as input, and uses fully connected layers to generate a 64-dimensional feature vector for face and eye position representations. Face-Net uses a convolutional network stacked with several SELayers to convert an input face image into a 64-dimensional feature vector. The two 64-dimensional features are concatenated as facial feature guidance for gaze estimation. Eye-Net takes the left and right eye images as inputs for the two-stream collaborative architecture, and processes the images using the proposed CCBs under the guidance of facial features and channel-mixing layers to learn a 128-dimensional feature representation. Gaze point regression and classification results are predicted from the feature representation using fully connected layers.

### CCB

Existing self-attention models for gaze estimation primarily rely on the fusion of the eye features through assigning channel-wise attention weights based on facial features to each eye. However, in the gaze-estimation task, one may need both eyes to provide collaborative attention. Therefore, the CCB is proposed and iteratively applied to correlate eye contexts at different depths.

Particularly, following the concepts in self-attention, for the left and right eye features X_{l,r}_ ∈ R^h×w×c^ at the same depth-level of the network, queries are defined as Q_{l,r}_ = X_{l,r}_, keys as K_{l,r}_ = X_{l,r},_ and values as V_{l,r}_ = X_{l,r}_W_v_, where W_v_ is the embedding matrix shared between eyes, implemented as 1 × 1 convolution. CCB first computes the contextual representation $K^\ast_{l/r}$ ∈ R^h×w×c^ for each eye with 3 × 3 group convolutions over all the neighboring keys within a 3 × 3 grid. Subsequently, the query and contextual representation for each eye are concatenated and two 1 × 1 convolutions, W_α_ and W_β,_ that share weights between left and right eyes are used to learn corresponding correlated attention matrix:

1 $${\mathrm{A}}_{\mathrm{l}/\mathrm{r}}=\left[{\mathrm{K}^{*}}_{{\mathrm{l}}/\mathrm{r}},{\mathrm{Q}}_{\mathrm{l}/\mathrm{r}}\right]{\mathrm{W}}_{\mathrm{\alpha }}{\mathrm{W}}_{\mathrm{\upbeta }}$$

**Fig. 3:**
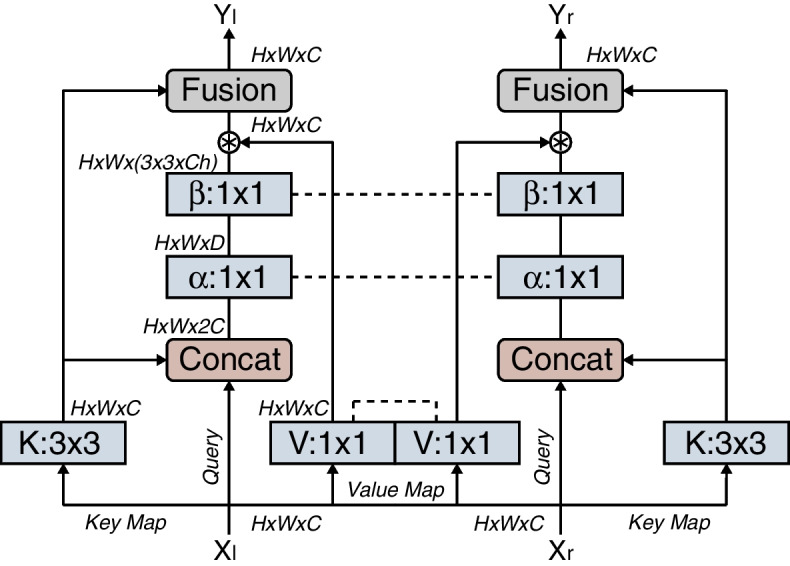
Structure of the proposed CCB. It correlates two features using the contextual attention mechanism. Please refer to the text for details

All values are then aggregated to compute the correlated representation for each eye as $K^\prime_{l/r}=V^\ast_{l/r}$ ⊛ $A_{l/r}$ , where ⊛ denotes the non-local operator originally investigated by [46]. The final output feature for each eye is a fusion of the correlated and contextual representations, $K^\prime_{l/r}$ and $K^\ast_{l/r}$, respectively, using selective kernels [47]. Figure 3 shows the structure of the CCB.

### Channel-mixing layers

Although some gaze-estimation methods, such as iTracker [2] and SAGE [3], directly concatenate facial and eye features and feed them into several fully connected layers, such a simple multi-layer perception (MLP) over concatenated features may lose long-distance communications between feature channels. To address this problem, MLP-Mixer [48] investigates a pure MLP architecture for vision tasks, which achieves similar or even better performance than Transformer. The core of MLP-Mixer is the patch-based feature transformation using a mixing operator:

2 $${\mathrm{Y}}_{\mathrm{c}}={\mathrm{X}}_{\mathrm{c}}+{\mathrm{W}}_{2}\cdot \upsigma ({\mathrm{W}}_{1}\cdot \mathrm{LayerNorm}(\mathrm{Xc}))$$

where X and Y denote the input and output features, c denotes a feature channel, σ denotes an activation function, and W_1_ and W_2_ are weights. This mixing operator can capture long-distance channel communications. Inspired by this, this study uses four channel-mixing layers before the final fully connected layer, to fuse the eye and facial features and compute feature embedding.

### Loss function

According to ref. [49], from a maximum likelihood estimation perspective, a neural network can be simultaneously optimized by mean square error loss and cross-entropy loss along a coherent direction. Inspired by this, this study incorporates the gaze-classification task as additional supervision for gaze estimation, where quadrant divisions of a screen are adapted, particularly for common gaze datasets. Thus, the proposed method is robust and can be extended to any division. Specifically, given a set of training images X = {x_i_}^N^_i=1_, and corresponding ground truth gaze labels Z = {y_i_, q_i_ }^N^_i=1_, where N is the number of training images, this study aims to learn a mapping function modeled by a neural network by minimizing the regression spatial offset between the predicted 2D gaze point yˆ_i_ and ground truth y_i_. Furthermore, the classification error typically formulated as a softmax loss should be minimized:

3 $$L_s=\frac{1}{N}\sum_{i=1}^{N}-log\frac{{e}^{{f}_{{q}_{i}}}}{{\sum }_{j=1}^{C}{e}^{{f}_{j}}}$$

where C is the class number, f _j_ indicates that the input feature is compressed for a label j through a fully connected layer with weights W_j_ as f _j_ = W_j_x_i_.

In the proposed method, the large margin cosine loss [36]—a state-of-the-art metric learning model—is used to supervise the classification. It is derived from a normalized version of softmax loss, by fixing ||W_j_|| = 1 using L_2_ normalization and ||x|| = s such that:

4 $${\mathrm{f}}_{\mathrm{j}}={\mathrm{W}}_{\mathrm{j}}{\mathrm{x}}_{\mathrm{i}}=||{\mathrm{W}}_{\mathrm{j}}|| ||{\mathrm{x}}_{\mathrm{i}}||\mathrm{ cos}({\mathrm{\theta }}_{\mathrm{j},\mathrm{i}})=\mathrm{s cos}({\mathrm{\theta }}_{\mathrm{j},\mathrm{i}})$$

where s = 64 is a constant, θ _j,i_ is the angle between W_j_ and x_i_. For two classes C_i _(i = 1, 2), conditions cos θ_1_ > cos θ_2_ for C_1_ and cos θ_1_ < cos θ_2_ for C_2_ are guaranteed for correct classification. A fixed margin *m* is introduced to improve the discrimination between features by ensuring cos θ_1_ −m > cos θ_2_ for C_1_, and vice versa. With this technique, large margins in the cosine space are encouraged for feature-embedding optimization. For more details, please refer to ref. [36].

From EM-Gaze, a 128-dimensional gaze-feature embedding is optimized using a large margin cosine loss, L_lmc_, which is associated with the four-quadrant division-based classification, defined as:

5 $${L}_{lmc}=\frac{1}{N}\sum\nolimits_{i=1}^{N}-log\frac{{e}^{s\left(\mathrm{cos}\left({\theta }_{{q}_{i}},i\right)-m\right)}}{{e}^{s\left(\mathrm{cos}\left({\theta }_{{q}_{i}},i\right)-m\right)}+\sum_{j\ne {q}_{i}}{e}^{scos({\theta }_{j,i})}}$$

where *m* = 0.4 is the fixed large margin in cosine space.

EM-Gaze outputs a 2D vector yˆ_i_ using a fully connected layer, supervised by a smooth L_1_ regression loss:

6 $${L}_{reg}=\left\{\begin{array}{c}0.5{\left(\widehat{{y}_{i}}-{y}_{i}\right)}^{2},\left|\widehat{{y}_{i}}-{y}_{i}\right|\le 1\\ \left|\widehat{{y}_{i}}-{y}_{i}\right|-0.5, otherwise\end{array}\right.$$

The overall loss function is defined as: L = L_lmc_ + λ L_reg_, where λ = 150 is a constant parameter that balances the loss terms.

### Implementation details

The inputs to EM-Gaze are a face image (224 × 224 × 3), two eye images (112 × 112 × 3) with the right eye image flipped, and 12-dimensional eye and face bounding box corners.

Label-Net stacks four fully connected layers whose output channels are 64, 96, 128, and 64, respectively. Face-Net consists of six convolutional layers; the numbers of convolutional kernels are 48, 96, 128, 192, 128, and 64; the kernel sizes of the first three layers are 5 and the remainder are 3, and the strides are 2, 1, 1, 1, 2, and 2, respectively. Each convolutional layer is followed by group normalization and ReLU activation function, and a 3 × 3 max pooling layer is applied after the second and third convolutional layers. SELayers are added after the second, fourth, and last convolutional layers. Two fully connected layers follow the convolutional layer to further compress the face feature to a 64-dimensional vector.

Eye-Net has five convolutional layers, the numbers of convolutional kernels are 24, 48, 64, 128, and 64; the kernel sizes of the first three layers are 5 and the remainder are 3, and the strides are 2, 1, 1, 1, 2, and 2. Group normalization, activation, max pooling, and SELayers have the same settings as that of Face-Net. The last four convolutional layers are fused with facial features by AdaGN and correlated by the proposed CCB. A fully connected layer then converts the two-eye features to a 128-dimensional vector. The eye and facial features are concatenated and fed into two fully connected layers to produce a 128-dimensional vector. The vector is then fed into the following four channel-mixing layers to output the same dimensional gaze feature. Finally, one fully connected layer follows to convert the feature to a two-dimensional vector for 2D gaze point regression and the other converts it to a four-dimensional vector for gaze point classification on quadrant divisions.

The learning rate for training EM-Gaze is set to 0.001 and half-reduced after every eight epochs. The batch size is set to 256. The proposed network is trained in 50 epochs and its weights are initialized using default Xavier initialization [50]. Similar to AFF-Net [4], face and eye bounding boxes are made to randomly move less than 30 pixels to improve model robustness during training. EM-Gaze is implemented using PyTorch [51], and the weights of all layers are initialized using the default initialization.

## Results and discussion

This section presents experimental results, including comparisons with state-of-the-art deep learning-based gaze-estimation methods, an ablation study of the proposed techniques, and additional analysis of public datasets.

### Datasets and evaluation metrics

The experiments are conducted using two popular gaze-estimation datasets: GazeCapture dataset [2] and MPIIFaceGaze [31]. The GazeCapture dataset is the largest unconstrained gaze dataset captured by mobile devices. It collects face images and corresponding 2D gaze data through crowdsourcing with 2445504 images from 1474 subjects. The dataset is captured by the front-facing camera of mobile phones or tablets, by asking the subjects to look at randomly generated points on the screen while recording the coordinates and full-face images. Additionally, the GazeCapture dataset provides the meta-data of display size and camera position, such that the quadrant division label can be computed for a gaze point. This study follows the same train and tests data split as ref. [2] by taking 150 subjects for testing and the remainder for training. The MPIIFaceGaze dataset is the largest gaze-estimation dataset for 3D gaze and serves as a common benchmark for appearance-based methods. It contains over 200000 images from 15 subjects and provides a standard evaluation tool. The methods are tested on the standard evaluation set, which contains 3000 testing images from each subject.

### Data processing

Regarding the GazeCapture dataset, face and eye images are cropped based on corresponding bounding boxes detected through an open-sourced python face-recognition library. Face and eye images are resized to 224 × 224 × 3 and 112 × 112 × 3, respectively. Additionally, the right eye image is flipped as AFF-Net [4] does, which was proven to be effective in improving accuracy. Regarding the MPIIFaceGaze dataset, the data-processing instruction by ref. [9] is followed to obtain the face and eye bounding boxes. The image is cropped and resized using the same settings as that used for the GazeCapture dataset. The bounding boxes are represented by bottom-left and top-right corner values, normalized with respect to the image sizes. Finally, to simulate the calibration-free settings, the leave-one-person-out test is performed and the results are averaged from all subjects as the final performance for a method on the MPIIFaceGaze dataset.

### Evaluation metrics

Regarding gaze point prediction, the Euclidean distance error between the ground truth and estimated gaze point on the screen in physical distance is reported. Regarding quadrant division-based classification, the Top-1 accuracy on the four labels, which denote the four quadrants divided by the center point, is reported. For fair comparisons, only statistical results of calibration-free methods are reported.

### Comparison with appearance-based methods

The proposed method is compared with other appearance-based methods on both the GazeCapture and MPIIFaceGaze datasets.

On the GazeCapture dataset, the proposed method is evaluated against four representative methods, which are iTracker [2], SAGE [3], TAT [52], and AFF-Net [4]. The open-source code released by the authors is used to test iTracker and AFF-Net. Considering that SAGE and TAT can be improved by introducing multiple calibration images, only the results from SAGE and TAT without extra calibration are shown for fair comparison, and the gaze point regression values based on the papers are reported. Table 1 displays the gaze point regression and classification performances for phones and tablets, respectively. Regarding performances on phones, iTracker has the largest regression error of 2.06 cm. SAGE and TAT have similar performances of approximately 1.77 cm. AFF-Net improves the result to 1.62 cm, and the proposed method achieves an error of 1.57 cm. On tablet devices, the regression errors for iTracker, SAGE, TAT, and AFF-Net are 3.22, 2.72 2.66, and 2.30 cm respectively. The proposed method outperforms the other methods and achieves a 2.21 cm regression error. For the classification metric, EM-Gaze achieves 12.1% and 15% improvements over the second-best methods on phones and tablets respectively.

**Table 1:** Gaze regression and classification results on the GazeCapture dataset. For SAGE [3] and TAT [52] methods, classification results are unavailable from publicly accessible contents. EM-Gaze outperforms the alternative methods under gaze-regression error and Top-1 classification accuracy metrics

Method	Phone		Tablet	
	Error ↓ (cm)	Accuracy ↑ (%)	Error ↓ (cm)	Accuracy ↑ (%)
iTracker [2]	2.06	51.9	3.22	65.8
SAGE [3]	1.78	-	2.72	-
TAT [52]	1.77	-	2.66	-
AFF-Net [4]	1.62	57.1	2.30	70.1
**EM-Gaze (ours)**	**1.57**	**69.2**	**2.21**	**85.1**

More experiments are conducted on the MPIIFaceGaze dataset. Considering that the MPIIFaceGaze dataset is a commonly used 3D gaze-estimation dataset, both the Euclidean distance and converted 3D angle errors based on provided camera-screen calibration matrix are shown. Note that the MPIIFaceGaze dataset is collected from a laptop without access to the physical center point of the display; thus, only regression errors are reported and classification and metric learning are not used as supervision for the EM-Gaze method. iTracker, Spatial weights CNN [31], RT-GENE [23], and AFF-Net were selected as the competitive methods. As shown in Table 2, the proposed method exhibits state-of-the-art performances over the other methods on the MPIIFaceGaze dataset, with a 3.60 cm Euclidean distance error and a 4.10 cm angular error.

**Table 2:** Gaze regression results on the MPIIFaceGaze dataset. The angular error for 3D gaze is converted from 2D gaze through post-processing

Method	2D Gaze ↓ (cm)	3D Gaze ↓ (deg)
iTracker [2]	5.46	6.20
Spatial weights CNN [31]	4.20	4.80
RT-GENE [23]	4.20	4.80
AFF-Net [4]	3.90	4.40
**EM-Gaze (ours)**	**3.60**	**4.10**

Comparisons with representative appearance-based methods on the GazeCapture and MPIIFaceGaze datasets demonstrate a clear advantage of EM-Gaze over the other methods.

### Ablation study

To demonstrate the effectiveness of the CCB, channel-mixing layer, and metric-learning strategy, an ablation study is performed on the GazeCapture dataset.

### Ablation study about components

The effectiveness of the CCB and channel-mixing layer is demonstrated. As aforementioned, CCB correlates two eye features during feature extraction, and the channel-mixing layer fuses eye and facial features to generate feature embedding. Table 3 presents the experimental results without and with CCB or channel-mixing layer modules on the GazeCapture dataset under the Euclidean distance metric; metric-learning strategy is disabled. The proposed network with CCB and channel-mixing layer modules achieves average errors of 1.59 and 2.22 cm on phones and tablets, respectively. Without the channel-mixing layer, the results degenerate to 1.60 and 2.27 cm on phones and tablets, respectively; without CCB, the results further degenerate to 1.61 and 2.29 cm on phones and tablets, respectively. The original network without CCB or the channel-mixing layer performed worst.

**Table 3:** Ablation study of CCB and channel-mixing layer on the GazeCapture dataset. Gaze regression performances are reported

CCB	Channel-mixing layer	GazeCapture	
		Phone (cm)	Tablet (cm)
-	-	1.62	2.30
-	✓	1.61	2.29
✓	-	1.60	2.27
✓	✓	**1.59**	**2.22**

### Ablation study about strategy

**Fig. 4:**
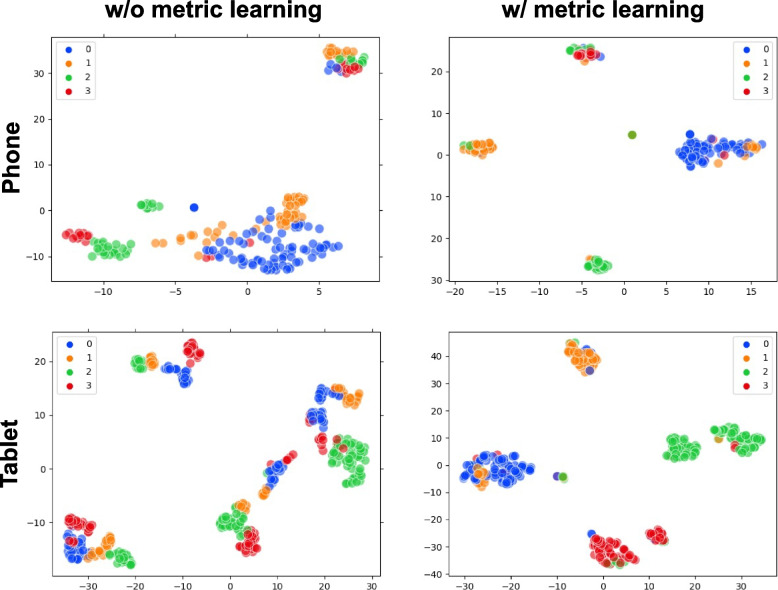
t-SNE visualizations of gaze-feature embedding without and with metric learning. Top: feature embedding for a user on a phone; bottom: feature embedding for a user on a tablet. Each dot represents an embedded feature from an input face image; the color of the dot represents its ground truth class label

The effectiveness of the metric-learning strategy for gaze classification on quadrant divisions is further evaluated. Figure 4 shows t-SNE [53] visualizations of embedded features from face images of the same user without and with the metric-learning strategy for phones and tablets, respectively. The results reveal a significant difference before and after using metric learning. With metric learning, gaze features belonging to the same quadrant division are grouped more closely, and the gaps between different clusters are increased. The performances without and with L_lmc_ for iTracker, AFF-Net, and EM-Gaze methods are reported. Table 4 presents the results. With the metric-learning strategy, all methods achieve improved regression and classification performances except for AFF-Net, from which a degenerated regression performance is observed. EM-Gaze with metric learning exhibits the best performance among all the methods.

**Table 4:** Ablation study of the proposed metric-learning strategy on the GazeCapture dataset

Method	Phone		Tablet	
	Error ↓ (cm)	Accuracy ↑ (%)	Error ↓ (cm)	Accuracy ↑ (%)
iTracker w/o L_lmc_	2.11	51.9	3.41	65.8
iTracker w/ L_lmc_	2.08	63.4	3.30	79.0
AFF-Net w/o L_lmc_	1.63	57.1	2.35	70.1
AFF-Net w/ L_lmc_	1.65	68.4	2.46	83.2
**EM-Gaze (ours) w/o** L_lmc_	**1.59**	**57.9**	**2.22**	**71.0**
**EM-Gaze (ours) w/** L_lmc_	**1.57**	**69.2**	**2.21**	**85.1**

### Additional analysis

**Fig. 5:**
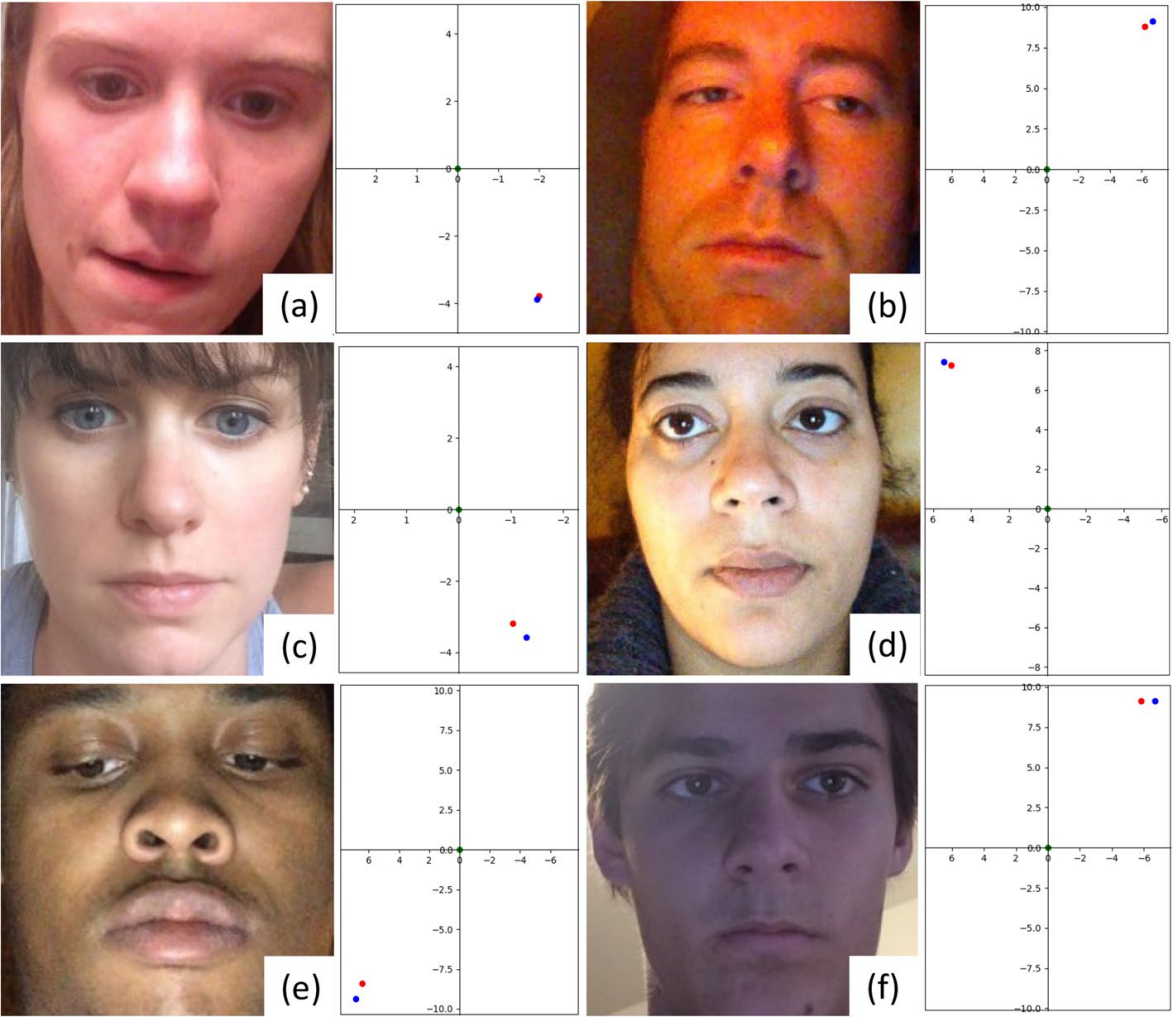
Representative face images and corresponding gaze-estimation results from the GazeCapture dataset. Red and blue dots indicate the estimated and ground truth gaze points, respectively

Figure 5 shows the representative visual results of face images and corresponding gaze point predictions from EM-Gaze. The proposed method performs well under various lighting (Fig. 5a, c, e) and head pose (Fig. 5b, d, f) conditions.

Inference run-time performance of EM-Gaze and state-of-the-art methods are reported on a single RTX 2080Ti GPU. The inference speed of EM-Gaze is 136 FPS, significantly faster than iTracker. RT-GENE and AFF-Net run slightly faster than EM-Gaze. Regarding model size, EM-Gaze has 2.7M parameters, which is slightly more than that of AFF-Net, and is twice smaller than that of iTracker and significantly smaller than that of RT-GENE. Table 5 lists corresponding statistics. In summary, EM-Gaze has a good balance of model size and efficiency to estimate accurate gaze on mobile devices.

**Table 5:** Run-time performance and model size statistics

Method	FPS ↑	Params (M) ↓
iTracker	28	6.3
RT-GENE	170	31.7
AFF-Net	156	1.9
EM-Gaze (ours)	136	2.7

## Conclusions

This study proposed EM-Gaze for mobile gaze estimation, including gaze point regression and classification on quadrant division of the display. EM-Gaze efficiently correlated eye contexts, fused channels for long-distance communications, and used metric learning to optimize gaze-feature embedding. The experimental results indicated that EM-Gaze achieves state-of-the-art gaze-estimation performance on the GazeCapture and MPI-IFaceGaze datasets.

**Fig. 6:**
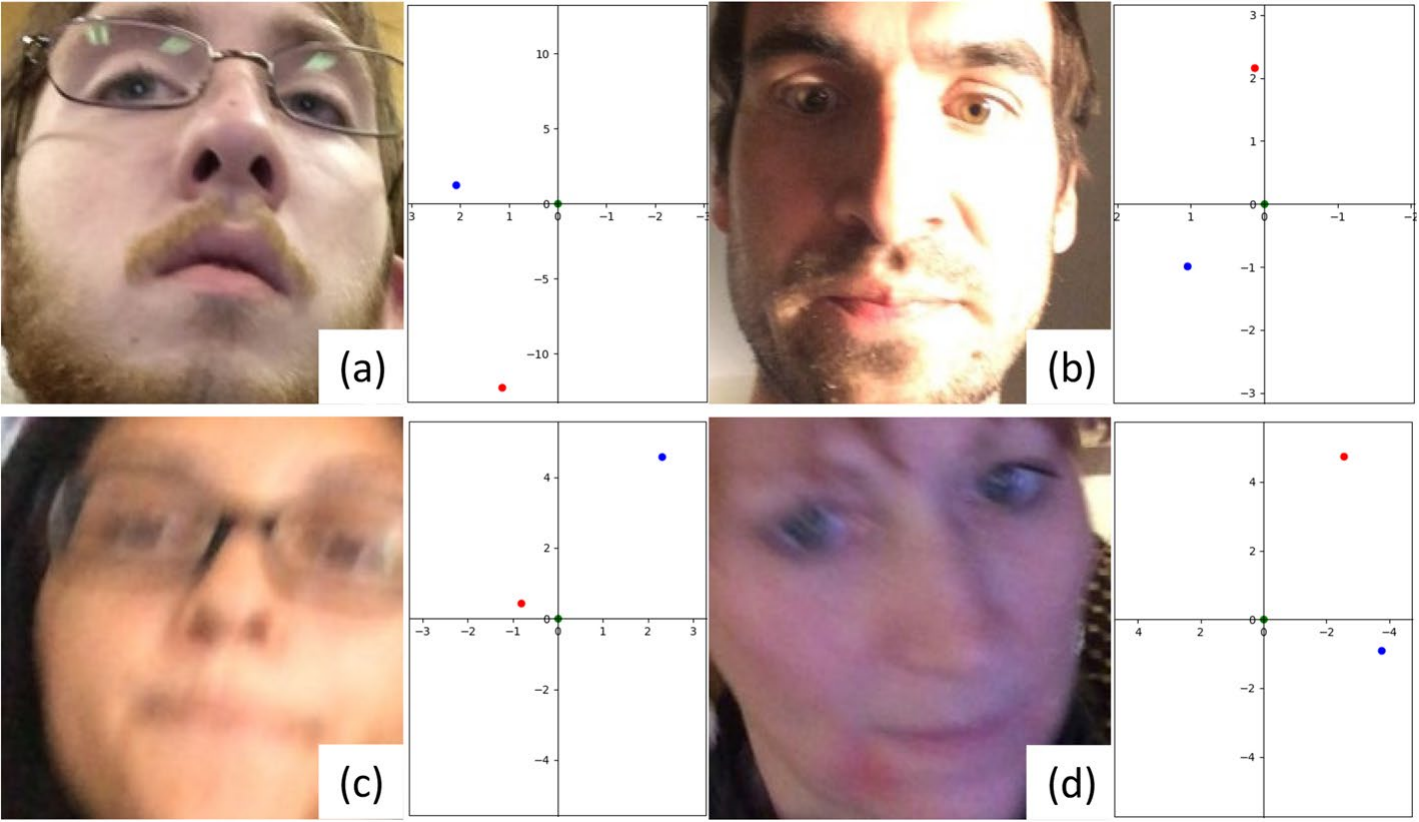
Failure cases. **a** tilted head pose; **b** inconsistent lighting; **c** and **d** motion blur. Red and blue dots indicate the estimated and ground truth gaze points, respectively

EM-Gaze could fail to predict correct gazes for challenging inputs. First, when the head pose is overly tilted, EM-Gaze may fail because of the strong impact imposed by the head pose. Second, inconsistent lighting on the face can disturb the prediction. Third, motion blurs existed in the testing data, which made the prediction fail. Figure 6 shows representative failure cases.

In the future, disentangled representation of the face, including geometry, appearance, and illumination, will be studied to alleviate the distraction caused by inconsistent lighting, motion blur, etc. Furthermore, introducing few-shot calibration into the proposed architecture is a promising research direction.

## Data Availability

The data that support the findings of this study are available from the corresponding author upon request.

## Abbreviations

CCB: Context correlation block
MLP: Multi-layer perception
NSL: Normalized version of softmax loss
AdaGN: Adaptive group normalization
L-Softmax: Large margin softmax
